# High SARS‐CoV‐2 Exposure in Rural Southern Mozambique After Four Waves of COVID‐19: Community‐Based Seroepidemiological Surveys

**DOI:** 10.1111/irv.13332

**Published:** 2024-06-05

**Authors:** Áuria de Jesus, Rita Ernesto, Arsénia J. Massinga, Felizarda Nhacolo, Khátia Munguambe, Alcido Timana, Arsénio Nhacolo, Augusto Messa, Sérgio Massora, Valdemiro Escola, Sónia Enosse, Rufino Gunjamo, Carlos Funzamo, Jason M. Mwenda, Joseph Okeibunor, Alberto Garcia‐Basteiro, Caterina Guinovart, Alfredo Mayor, Inácio Mandomando

**Affiliations:** ^1^ Centro de Investigação em Saúde de Manhiça (CISM) Maputo Mozambique; ^2^ Faculdade de Medicina Universidade Eduardo Mondlane (UEM) Maputo Mozambique; ^3^ Instituto Nacional de Saúde (INS) Ministério da Saúde Marracuene‐Maputo Mozambique; ^4^ Mozambique Country Office World Health Organization Maputo Mozambique; ^5^ Regional Office for Africa (AFRO) World Health Organization Brazzaville Republic of Congo; ^6^ ISGlobal, Hospital Clínic–Universitat de Barcelona Barcelona Spain; ^7^ Amsterdam Institute for Global Health and Development Academic Medical Centre Amsterdam The Netherlands; ^8^ Department of Physiologic Sciences, Faculty of Medicine Universidade Eduardo Mondlane Maputo Mozambique

**Keywords:** antibodies, COVID‐19, Mozambique, SARS‐CoV‐2, seroprevalence, serosurvey, sub‐Saharan Africa

## Abstract

**Background:**

Mozambique was one of many African countries with limited testing capacity for SARS‐CoV‐2. Serosurveys, an alternative to estimate the real exposure to understand the epidemiology and transmission dynamics, have been scarce in Mozambique. Herein, we aimed to estimate the age‐specific seroprevalence of SARS‐CoV‐2 in the general population of the Manhiça District, at four time points, for evaluating dynamics of exposure and the impact of vaccination.

**Methods:**

We conducted four community‐based seroepidemiological surveys separated by 3 months between May 2021 and June 2022 to assess the prevalence of SARS‐CoV‐2 antibodies. An age‐stratified (0–19, 20–39, 40–59, and ≥ 60 years) sample of 4810 individuals was randomly selected from demographic surveillance database, and their blood samples were analyzed using WANTAI SARS‐CoV‐2 IgG + IgM ELISA. Nasopharyngeal swabs from a subsample of 2209 participants were also assessed for active infection by RT‐qPCR.

**Results:**

SARS‐CoV‐2 seroprevalence increased from 27.6% in the first survey (May 2021) to 63.6%, 91.2%, and 91.1% in the second (October 2021), third (January 2022), and fourth (May 2022) surveys, respectively. Seroprevalence in individuals < 18 years, who were not eligible for vaccination, increased from 23.1% in the first survey to 87.1% in the fourth. The prevalence of active infection was below 10.1% in all surveys.

**Conclusions:**

A high seroprevalence to SARS‐CoV‐2 was observed in the study population, including individuals not eligible for vaccination at that time, particularly after circulation of the highly transmissible Delta variant. These data are important to inform decision making on the vaccination strategies in the context of pandemic slowdown in Mozambique.

## 1 Background

The spread of the COVID‐19 pandemic has shown different epidemiological, virological, and clinical dynamics for each continent and country. SARS‐CoV‐2 rapidly spread all over the world, and by April 2023, over 540 million cases and around 6 million deaths have been recorded, with an unprecedented impact on the global economy [1]. Unexpectedly, in contrast to high‐income countries, which accounted for approximately 70.7% of confirmed cases and associated mortality worldwide, the African countries, with the exception of South Africa, reported relatively low numbers [2]. Limited testing capacity due to financial constraints, challenges on global procurement of diagnostic tests, and testing criteria or health‐seeking behavior may have contributed to this lower impact in the African continent, although factors have not been fully elucidated [3].

Asymptomatic carriers are important reservoirs and source of transmission of many respiratory infectious disease, and COVID‐19 is not an exception. In the context of limited testing capacity, community serological surveys that measure antibodies across populations are used to provide insights into the epidemiology and transmission of different pathogens including SARS‐CoV‐2 [4, 5, 6, 7]. These serosurveys can give an estimation of the proportion of individuals that have been exposed to SARS‐CoV‐2, providing critical data to inform decision making on control strategies such as vaccination [5]. This approach has been extensively used for SARS‐CoV‐2 globally and especially in African countries [3, 8, 9, 10] where testing capacity was an issue, under the coordination of the World Health Organization (WHO), in the UNITY protocol [11].

Mozambique, similarly to other African countries, has a fragile health system; however, the impact of the COVID‐19 pandemic was unexpectedly low. As of March 2023, around 230,000 confirmed cases and around two thousand deaths (representing 0.03% of the global deaths) were reported. The country experienced four epidemic waves [12], dominated by the wild‐type SARS‐CoV‐2, Beta, Delta, and Omicron variants during the first (September–December 2020), second (January–March 2021), third (June–September 2021), and fourth (December 2021–January 2022) waves [13, 14]. Following global recommendations, Mozambique introduced vaccination against SARS‐CoV‐2, one year after the first confirmed case through the COVAX mechanism and with financial support from international donors [15, 16]. Three vaccine formulations were used, including (i) the inactivated COVID‐19 vaccine BIBP (aluminum‐hydroxide‐adjuvanted, inactivated whole virus vaccine) developed by China National Biotec Group (CNBG), China National Pharmaceutical Group corporation (Sinopharm); (ii) the Ad26.COV2.S vaccine (recombinant, replication‐incompetent adenovirus serotype 26—Ad26 vector encoding a full‐length and stabilized SARS‐CoV‐2 spike protein), manufactured by Janssen (Johnson and Johnson); and (iii) the ChAdOx1‐S [recombinant] vaccine against COVID‐19 (AstraZeneca COVID‐19 vaccine AZD1222 Vaxzevria, SII COVISHIELD) developed by the Oxford University and AstraZeneca [17]. Vaccination was rolled out in different phases, with the first phase of vaccination prioritizing the most exposed and vulnerable groups (healthcare workers, individuals with underlying conditions and over 50 years old). The second phase expanded the target groups, including people above 30 years of age and, later, people above 18 years. As of July 2, 2023, the WHO indicates that 22,869,646 individuals were administered at least one dose of COVID‐19 vaccines in Mozambique, which corresponds to 74.2% of the Mozambican population in 2021 (30,832,244 individuals) [1, 18, 19].

The low number of reported cases, especially when considering the limited testing and surveillance capacity within the country, suggests a much larger but undetected impact of SARS‐CoV‐2 [20]. Additionally, data from national serosurveys conducted by the National Institute of Health (INS) between June and December 2020 suggest that, before the third wave, less than 8% of individuals had been exposed to SARS‐CoV‐2 [21]. Therefore, updated seroprevalence data are crucial to understand the transmission dynamics and epidemiology of SARS‐CoV‐2 in Mozambique. Herein, we aimed to estimate the age‐specific seroprevalence of SARS‐CoV‐2 in the general population of the Manhiça District, a semi‐rural area in southern Mozambique, at four different time points (four serosurveys) separated from each other by 2–3 months, to inform public health policy by evaluating the exposure dynamics and the impact of vaccination.

## 2 Materials and Methods

### 2.1 Study Site and Population

The study was conducted by the *Centro de Investigação em Saúde de Manhiça* (CISM), in six administrative posts (Maluana, 3 de Fevereiro, Xinavane, Calanga, Manhiça‐Sede, and Ilha Josina) in Manhiça District, located 80 km north of the country's capital city, Maputo, in southern Mozambique. The Manhiça district covers 2373 km^2^ and has a subtropical climate, with two distinct seasons: the warm and rainy season (November to April) and the cool and dry lasting for the rest of the year [22]. Two critical communication networks cross the town of Manhiça, namely, the National Road number 1 (connecting the south and north of country as well as with other southern African countries) and the railway from Maputo to Zimbabwe. Since 1996, CISM has been conducting a continuous Health and Demographic Surveillance System (HDSS), which currently follows 201,845 individuals living in 46,441 enumerated and geo‐positioned households with regular updates of all demographic events [23]. The HDSS study area includes 19 health centers and two hospitals where morbidity surveillance is routinely conducted [22].

### 2.2 Study Design

Four community‐based serosurveys were conducted between May 2021 and June 2022, lasting approximately 1–2 months each. The first survey was conducted from May 3 to June 18, 2021; the second from October 5 to November 24, 2021; the third from January 10 to February 18, 2022; and the last one in May 4, 2022, to June 30, 2022. The serosurveys were conducted after each of the COVID‐19 waves in Mozambique, starting from the second wave (January to March of 2021). For each survey, the goal was to enroll 1200 participants selected from the HDSS database, following a two‐stage sampling scheme. First, a list of potential participants consisting of one individual per household was selected by simple random sampling from the HDSS database. Then, a stratified sampling was employed on the resulting list to select the study participants, with strata defined by same‐size (300 participants) age groups (0–19, 20–39, 40–49, ≥ 60 years); the sample size of 300 individuals per age group to be recruited was in order to estimate an age‐specific seroprevalence of 10%–90% with 3%–5% precision and 95% confidence level. In each of the four community‐based serosurveys, unique individuals were selected from the HDSS database with no overlap of individuals between surveys, with those randomly selected in one survey being excluded in the subsequent ones. For each potential participant, a list of 10 backup participants from the 10 closest households, matched by age group and sex, was provided for replacement in the case of refusals or absences. An individual was considered absent after three unsuccessful visits or phone calls. Selected individuals were visited at home, and informed consent (also informed assent for under age individuals who were 12 years or older) was obtained after checking inclusion criteria (be a resident of Manhiça registered in the HDSS). A standardized questionnaire to collect sociodemographic and clinical data was administered by a trained fieldworker, and blood samples were collected. Additionally, a subsample of 600 participants was randomly selected from each survey to estimate the prevalence of active SARS‐CoV‐2 infection by collecting nasopharyngeal (NP) swabs. The COVID‐19 vaccination status was based on self‐reporting and those who had received at least one dose where considered vaccinated.

This study is aligned with the WHO's Unity Studies standardized seroprevalence methods and protocol [4, 24].

### 2.3 Sample Collection

Five hundred microliters of capillary blood was collected by finger prick into an EDTA microtainer (Vacucare, EDTA.K3, Chennai, India) and stored in a cooler box containing ice packs until transportation to CISM's laboratory. Samples were centrifuged at 3500 rpm for 15 min, and plasma was collected into a clean tube (two aliquots per participant) and stored at −80°C until processing. NP swabs were collected following the Mozambican Ministry of Health guidelines for the detection of active SARS‐CoV‐2 infection by RT‐qPCR [25].

### 2.4 Detection of anti‐SARS‐CoV‐2 Antibodies

To determine the prevalence of total antibodies (IgM + IgG) against SARS‐CoV‐2, all plasma samples were analyzed using a commercially available Enzyme‐Linked Immunosorbent Assay (ELISA), the WANTAI SARS‐CoV‐2 Ab ELISA kit that detects antibodies against the receptor‐binding domain (RBD) of SARS‐CoV‐2 spike protein. Three negative controls and two positive controls (96.7% of sensitivity and 97.5% of specificity) were included in each assay, following the manufacturer's instructions [26]. Sample absorbance was read in a PowerWave XS ELISA plate reader (BioTek Instruments, Winooski, VT, USA, cat. no. MQX200R) controlled by the KC Junior software (BioTek Instruments, 1998‐2003). The cutoff (CO) for positivity was calculated based on the three controls included in the kit [27] using the following formula: CO = mean of three negative calibrator (NC) absorbance (Ab) + 0.16. If NC < 0.03, the threshold was set at 0.03. Sample Abs were converted to qualitative results by the following criteria: not detected if Ab/CO < 1, detected if Ab/CO ≥ 1, and unsure if Ab/CO = 0.9–1.1, in which case the results were considered invalid and were retested [26].

### 2.5 Data Capture and Statistical Analysis

Data were captured directly into an electronic questionnaire (REDCap software, Version 12.0.29) [28, 29] using tablets and were verified and corrected daily. Laboratory data were entered in the Servolab software (Servolab, Germany).

Frequencies and proportions were used to summarize categorical variables and 95% confidence intervals (95% CI) computed, and differences of proportions between age groups, vaccination status, serosurveys, and administrative posts were compared using the χ^2^ or Fisher's exact test when appropriate. Continuous variables were summarized using central tendency and dispersion measures. Statistical analyses were performed using R software Version 4.1.1.

### 2.6 Ethics Statement

The study protocol, data collection tools, and informed consent were reviewed and approved by the Mozambican National Bioethics Committee for Health (CNBS; Ref: 586/CNBS/20) and by CISM's Institutional Bioethics Committee for Health (CIBS; Ref: CIBS‐CISM/047/2020). After an explanation of the study objectives, characteristics, and procedures, each participant (or their parent/guardian in the case of individuals under 18 years) signed a written informed consent (and an informed assent for under age individuals who were 12 years or older) prior to enrollment and sample collection. An independent witness was also present and signed the consent (also the informed assent if applicable) if the participant or parent/guardian was illiterate. All personal data and information were anonymized or de‐identified in the electronic database.

## 3 Results

### 3.1 Description of Study Population

During the four serosurveys, 5656 participants were approached, and 85.1% (4810) were enrolled and provided blood samples, whereas the remaining 14.9% refused to participate in the study (Table 1). A total of 1323 (27.5%) blood samples did not have enough volume for testing and therefore were excluded from the analysis. Additionally, a subsample of 2209 enrolled participants also provided NP samples. The median age of the study participants ranged from 38.4 (IQR: 19.9–58.4) to 44.5 years (IQR: 26.2–62.9) in the first and last serosurveys, respectively (Table 1). Female participants were enrolled more frequently in all serosurveys (always more than 60% of the total); however, the sample size for each age group was balanced.

**TABLE 1:** Characteristics of participants enrolled in the four SARS‐CoV‐2 serosurveys in Manhiça district, southern Mozambique.

	Serosurvey 1 (*N* = 666)	Serosurvey 2 (*N* = 936)	Serosurvey 3 (*N* = 768)	Serosurvey 4 (*N* = 1117)
	*n* (%)	*n* (%)	*n* (%)	*n* (%)
Sex				
Male	214 (32.1)	312 (33.3)	246 (32.0)	325 (29.1)
Female	452 (67.9)	624 (66.7)	522 (68.0)	792 (70.9)
Pregnant women^a^	22/263 (8.4)	20/329 (6.1)	12/275 (4.4)	12/301 (4.0)
Age in years, median (IQR)	38.4 (19.9–58.4)	40.9 (20.2–59.7)	41.7 (21.7–58.7)	44.5 (26.2–62.9)
Age groups				
0–19 years	168 (25.2)	232 (24.8)	178 (23.2)	225 (20.1)
20–39 years	181 (27.2)	230 (24.6)	180 (23.4)	245 (21.9)
40–59 years	157 (23.6)	244 (26.1)	223 (29.0)	325 (29.1)
≥ 60 years	160 (24.0)	230 (24.6)	187 (24.1)	322 (28.8)
Administrative post				
Manhiça‐Sede	235 (35.3)	359 (38.4)	209 (27.2)	366 (32.8)
Maluana	123 (18.5)	135 (14.4)	129 (16.8)	158 (14.1)
Calanga	33 (4.9)	35 (3.7)	21 (2.7)	25 (2.2)
3 de Fevereiro	195 (29.3)	214 (22.9)	281 (36.6)	390 (34.9)
Ilha Josina Machel	23 (3.5)	88 (9.4)	50 (6.5)	64 (5.7)
Xinavane	57 (8.6)	105 (11.2)	78 (10.2)	114 (10.2)

Abbreviation: IQR = interquartile range.

^a^ Only women of reproductive age are considered (12–49 years).

### 3.2 Age‐Specific Seroprevalence of Antibodies Against SARS‐CoV‐2

**FIGURE 1:**
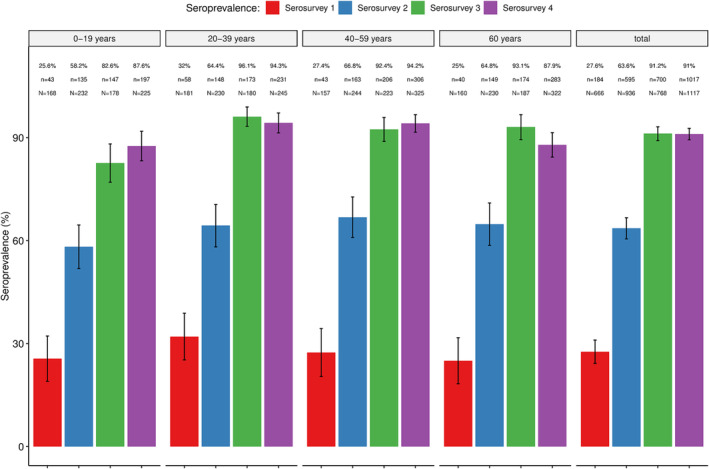
Overall and age‐specific seroprevalence of SARS‐CoV‐2 antibodies in four community‐based seroepidemiological surveys conducted in the district of Manhiça between May 2021 and June 2022. % = prevalence of seropositives; n = absolute number of positives per age group; N = total sample size per age group.

**FIGURE 2:**
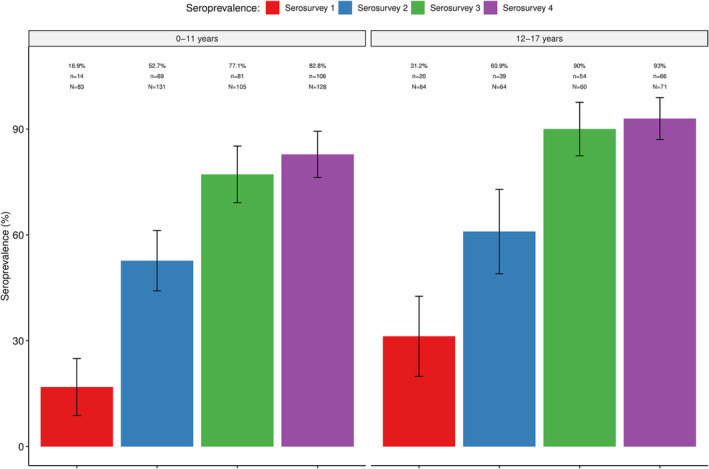
Seroprevalence of SARS‐CoV‐2 antibodies in four community‐based seroepidemiological surveys in age groups not eligible for vaccination. % = prevalence of seropositives; n = absolute number of positives per age group; N = total sample size per age group.

Antibodies (IgM and/or IgG) against SARS‐CoV‐2 were detected in all four serosurveys, with the seroprevalence increasing from 27.6% (95% CI: 24.2–31.0) in the first serosurvey to 63.6% (95% CI: 60.5–66.7) in the second and 91.2% (95% CI: 89.1–93.2) in the third. No statistically significant increases were observed between the third and fourth serosurvey (91.1%; 95% CI: 89.4–92.7). Similar trends were observed within the different age‐groups (Figure 1), including the youngest groups (0–11 years and 12–17 years) (Figure 2), which were not eligible for vaccination as per national recommendations (Tables S1 and S2). Overall, no statistically significant differences in seroprevalences between age‐groups were found, except in serosurveys three and four, which showed a lower seroprevalence in the age group of 0–19 years compared to the other age groups (Table S3).

### 3.3 Seroprevalence of SARS‐CoV‐2 Infections by Administrative Post

**FIGURE 3:**
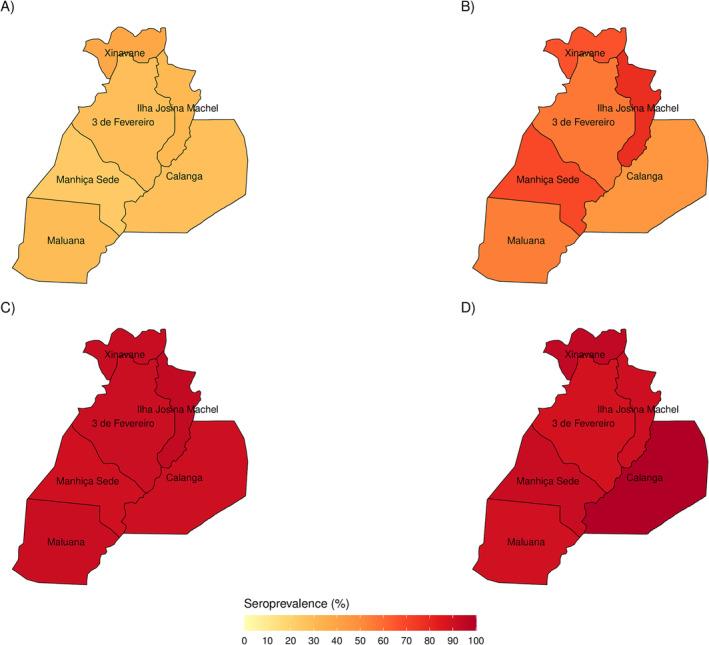
Seroprevalences in Manhiça district by administrative post and serosurvey. (A) Serosurvey 1 (May–June 2021). (B) Serosurvey 2 (October–November 2021). (C) Serosurvey 3 (January–February 2022). (D) Serosurvey 4 (May–June 2022).

The trend of increasing seroprevalence with time was observed in all administrative posts, as shown in Figure 3 and Table S4. Seroprevalences were highest in Xinavane (serosurvey 1), Ilha Josina (serosurveys 2 and 3), and Calanga (serosurvey 4). Calanga, a remote and isolated area, showed similar increases, reaching over 90% seroprevalence in serosurveys 3 (90.5%, 19/21) and 4 (100%, 25/25), although the number of recruited participants was relatively low compared to the other administrative posts. Manhiça‐Sede, the area with the highest population density, did not have the highest seroprevalence in any of the serosurveys.

### 3.4 Seroprevalence by Vaccination Status

**FIGURE 4:**
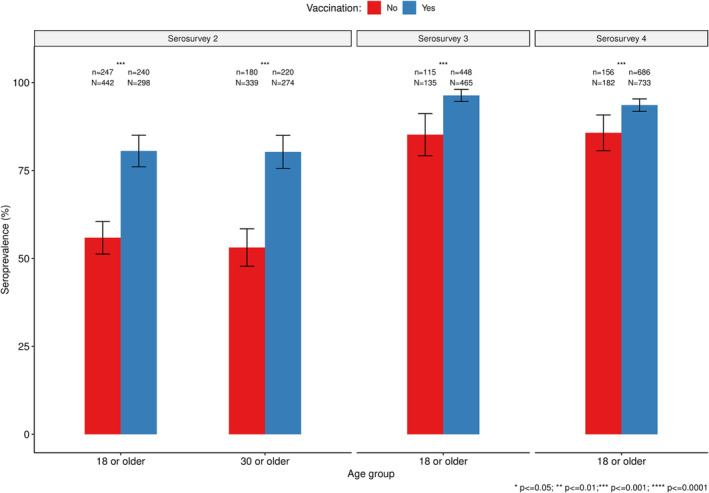
Comparison of the seroprevalence of SARS‐CoV‐2 antibodies between vaccinated and not vaccinated survey participants in Manhiça district.

To disentangle the contribution of vaccination and natural exposure to seropositivity, we performed a sub‐analysis to compare seroprevalence among individuals who had been vaccinated versus not vaccinated by serosurvey (Figure 4). The overall seroprevalence of SARS‐CoV‐2 antibodies was lower among unvaccinated individuals than in vaccinated ones (*p* ≤ 0.001) in the second, third, and fourth serosurveys. However, this difference became smaller in the third and fourth serosurveys, when approximately 85.2% (95% CI: 79.2–91.2) and 85.7 (95% CI: 80.6–90.8) of unvaccinated individuals had SARS‐CoV‐2 antibodies detected compared to 96.3% (95% CI: 94.6–98.0) and 93.6% (95% CI: 91.8–95.4) in the vaccinated group.

### 3.5 Prevalence of Active SARS‐CoV‐2 Infection Detected by PCR

The prevalence of RT‐qPCR‐detected SARS‐CoV‐2 infection was 3.8% (95% CI: 2.1–5.5), 0.7% (95% CI: 0.0–1.3), 10.1% (95% CI: 7.6–12.5), and 6.3% (95% CI: 4.3–8.2) in serosurveys 1, 2, 3, and 4, respectively. No statistically significant differences in prevalence of active infection between serosurveys or age groups were observed (Figure S1).

## 4 Discussion

This is the first study from Mozambique reporting seroprevalence data for SARS‐CoV‐2 from community‐based surveys conducted at different time points. Data suggest that seroprevalence increased very quickly from May 2021 to June 2022, with over 90% of the population in the district having been exposed to SARS‐CoV‐2 by January 2022 to February 2022 after the circulation of the Delta variant. The overall seroprevalence reported here increased between surveys, by two‐ and threefold within 3‐month intervals, mainly driven particularly by the Delta variant (more lethal) and maintained by the Omicron (more transmissible) variant, respectively [30, 31]. A similar trend was seen in individuals who were non‐eligible for vaccination, therefore ruling out the possibility that the increasing seroprevalence was due to vaccination.

The first serosurvey was conducted after the second wave in the country (January–March 2021), and it revealed seroprevalence levels around 30%, which is above the levels (0%–8%) reported from national serosurveys (June–December 2020) conducted prior to the second wave. However, it was not completely unexpected considering that during the second wave, the peak of positivity was 31.9% (January 2021) [21, 31]. The other serosurveys were implemented during or after the third and the fourth waves, which can explain the increasing trend in the seroprevalence.

Interestingly, the data from our seroepidemiological studies revealed that the most exposed age groups were those of participants aged 20–39 years (highest seroprevalences in the first, third, and fourth surveys) and 40–59 years (highest seroprevalence in the second and fourth surveys), although these differences were not significant. This is consistent with other reports from sub‐Saharan African countries that showed that those below 50 years were the most affected by SARS‐CoV‐2 infection [32]. This may be because this age group (< 50 years) is traditionally the most socioeconomically and socially active group. In addition, in the Manhiça District, young adults often migrate within (to and from the capital Maputo) and outside the country (most often South Africa)—thus the group most likely to be exposed to the virus, which is also consistent with other African reports [22, 23, 32, 33]. Another interesting finding is that in the second and third serosurveys, those participants aged ≥ 60 years had one of the highest seroprevalences (albeit the difference was only significant in the third survey with the 0–19 year age group) (Table S3); these surveys were conducted after the third and fourth COVID‐19 waves in the country. These results were different from what was reported in a meta‐analysis of the seroprevalence across the world, showing that the seroprevalences tended to decrease with age and seroprevalences rounded approximately 17% in the African population [33, 34]. One explanation for this difference may be the fact that some of these studies were conducted earlier in the pandemic (2020) though we report data starting from the second trimester of 2021. Another factor to consider is the prioritization of the population older than 50 years at the beginning of the vaccination in the country (the first phase started in March 2021) giving enough time for seroconversion to take place; therefore, a possible consequence is the higher levels of seroprevalence in this age group by the time of the second and third surveys (October 2021–February 2022). Furthermore, a final factor that can contribute for the seroprevalence of people ≥ 60 years to be on par with that of younger groups is that there are still some economically active individuals, who therefore are also exposed (possibly in public transportation and through contact with younger individuals in the same household). The PCR‐positive infection showed no association with seroprevalence levels.

In the first serosurvey, the seroprevalence varied by administrative post even though differences were not significant. However, seroprevalence increased rapidly over time in all administrative posts, including, unexpectedly, those with lower population density and in remote areas (Ilha Josina and Calanga) (Table S4) [35]. A factor that can explain the increase in the seroprevalence in these areas is the urban–rural movement that occurs daily within Manhiça and other parts of the province. Maputo City, which is the nearest city from Manhiça District (~80 km a part), has a large number of people moving daily mostly for work and access to essential services and goods; this was one of the cities with highest levels of prevalence in the country [22, 31]. Furthermore, the migration to and from southern African countries, particularly South Africa, has an important significance for the spread of the virus. The latter is especially relevant because a significant part of the Manhiça male adult population migrates to that country and travels back and forth regularly, mostly during the holiday season, and because of forced circumstances such as repatriations, medical needs, and others, also South Africa has been shown to be an important source for the introduction SARS‐CoV‐2 variants [13, 14].

As expected, the seroprevalence among vaccinated people was higher than in unvaccinated people, as vaccination induces antibody production and can also boost the immunity acquired due to natural infection [36]. Furthermore, the differences between these two groups tended to decrease with time, as more people were exposed during subsequent waves, particularly after the circulation of the more transmissible Delta (serosurveys 1 to 2) and Omicron (serosurveys 2 to 3) variants. This suggests that the seroconversion resulting from natural exposure can be as high and comparable with that resulting from vaccination. Nevertheless, this should be evaluated with caution as it was not possible to estimate antibody titers or the neutralizing ability of the antibodies detected. In addition, two additional factors should be considered: First, the group of participants that was eligible for vaccination changed over time, with an expansion to include all individuals who were at least 18 years old taking place while serosurvey 2 was underway. Second, given that the vaccination information was collected through self‐reporting, it is possible that some interviewees did not provide the real vaccination status; therefore, this analysis may be biased. This information will be crucial to support and manage the public health vaccination strategies referring specially to the unvaccinated and exposed population group.

This study has several limitations. First, seroprevalence data presented here refer to total antibodies (IgM and IgG) against only one antigen, and therefore, it was not determined if those antibodies are vaccine‐induced or acquired by natural exposure nor their neutralizing ability. Given that this information is crucial and will complement the findings presented in this study, additional analyses are underway and will be published separately. Second, given that the Manhiça District is semi‐rural with some semi‐urban areas and part of a national corridor (it is crossed by the main national road), with many migration movements, results might not be representative of other regions of the country which might have different population dynamics. Additionally, it is important to mention that the sample sizes for the sub‐analyses on the vaccination status by age groups are reduced, as these were sub‐sampled from the total population enrolled in the study, based on self‐reports on vaccination status; therefore, interpretations should take this into consideration.

## 5 Conclusion

A high seroprevalence to SARS‐CoV‐2 (over 90%) was observed in the study population, including individuals not eligible for vaccination at that time, particularly after the circulation of the highly transmissible Delta and Omicron variants suggesting that the impact of SARS‐CoV‐2 was underestimated. The data presented here were presented to the Mozambican health authorities and were considered in the decision‐making process for the adjustment of the vaccination strategies in the context of pandemic slowdown in Mozambique.

## Author Contributions

**Áuria de Jesus:** data curation, formal analysis, investigation, methodology, project administration, visualization, writing–original draft, writing–review and editing. **Rita Ernesto:** data curation, formal analysis, investigation, visualization, writing–original draft, writing–review and editing. **Arsénia J. Massinga:** data curation, formal analysis, investigation, methodology, project administration, software, visualization, writing–original draft, writing–review and editing. **Felizarda Nhacolo:** investigation, project administration, validation, writing–review and editing. **Khátia Munguambe:** conceptualization, investigation, methodology, supervision, validation, visualization, writing–review and editing. **Alcido Timana:** data curation, formal analysis, investigation, software, validation, visualization, writing–review and editing. **Arsénio Nhacolo:** data curation, formal analysis, investigation, methodology, software, visualization, writing–review and editing. **Augusto Messa Jr.:** formal analysis, investigation, visualization, writing–original draft, writing–review and editing. **Sérgio Massora:** supervision, validation, writing–review and editing. **Valdemiro Escola:** supervision, validation, writing–review and editing. **Sónia Enosse:** investigation, methodology, resources, supervision, writing–review and editing. **Rufino Gunjamo:** resources, supervision, writing–review and editing. **Carlos Funzamo:** funding acquisition, investigation, methodology, resources, supervision, writing–review and editing. **Jason M. Mwenda:** funding acquisition, methodology, supervision, writing–review and editing. **Joseph Okeibunor:** funding acquisition, methodology, supervision, writing–review and editing. **Alberto Garcia‐Basteiro:** conceptualization, investigation, methodology, visualization, writing–review and editing. **Caterina Guinovart:** conceptualization, methodology, supervision, visualization, writing–review and editing. **Alfredo Mayor:** conceptualization, funding acquisition, investigation, methodology, resources, supervision, validation, visualization, writing–original draft, writing–review and editing. **Inácio Mandomando:** conceptualization, funding acquisition, investigation, methodology, project administration, resources, supervision, visualization, writing–original draft, writing–review and editing.

## Conflicts of Interest

The authors declare no conflicts of interest.

### Peer Review

The peer review history for this article is available at https://www.webofscience.com/api/gateway/wos/peer‐review/10.1111/irv.13332.

## Supporting information


**Figure S1.** Age‐specific prevalence of SARS‐CoV‐2 PCR‐positive infection in the sub‐sample of participants that provided a nasopharyngeal swab in the four community‐based seroepidemiological surveys, Manhiça district.%: prevalence of seropositives; n:absolute number of positives per age group; N:total sample size per age group.

**Table S1.** Age‐specific seroprevalence of SARS‐CoV‐2 antibodies in 4 community‐based seroepidemiological surveys in Manhiça district, southern Mozambique.
**Table S2.** Seroprevalence of SARS‐CoV‐2 antibodies in 4 community‐based seroepidemiological surveys in Manhiça district, southern Mozambique in age groups not eligible for vaccination.
**Table S3.** Comparison of seroprevalence of SARS‐CoV‐2 antibodies between age groups in 4 community‐based seroepidemiological surveys in the Manhiça district, southern Mozambique.
**Table S4.** Evolution of the seroprevalence of SARS‐CoV‐2 antibodies by administrative post during 4 community‐based seroepidemiological surveys in Manhiça district.

## Data Availability

Deidentified individual‐level participant data and data dictionary will be available for sharing after approval of a proposal by the Manhiça Health Research Center (CISM) Internal Scientific Committee and following a signed data transfer agreement. Requests for data sharing can be directed to the corresponding author.
