# Supplementary figures and images for: High SARS‐CoV‐2 Exposure in Rural Southern Mozambique After Four Waves of COVID‐19: Community‐Based Seroepidemiological Surveys
Source: Influenza Other Respir Viruses. 2024 Jun 5;18(6):e13332. doi: 10.1111/irv.13332 (PMC11150860; doi:10.1111/irv.13332)

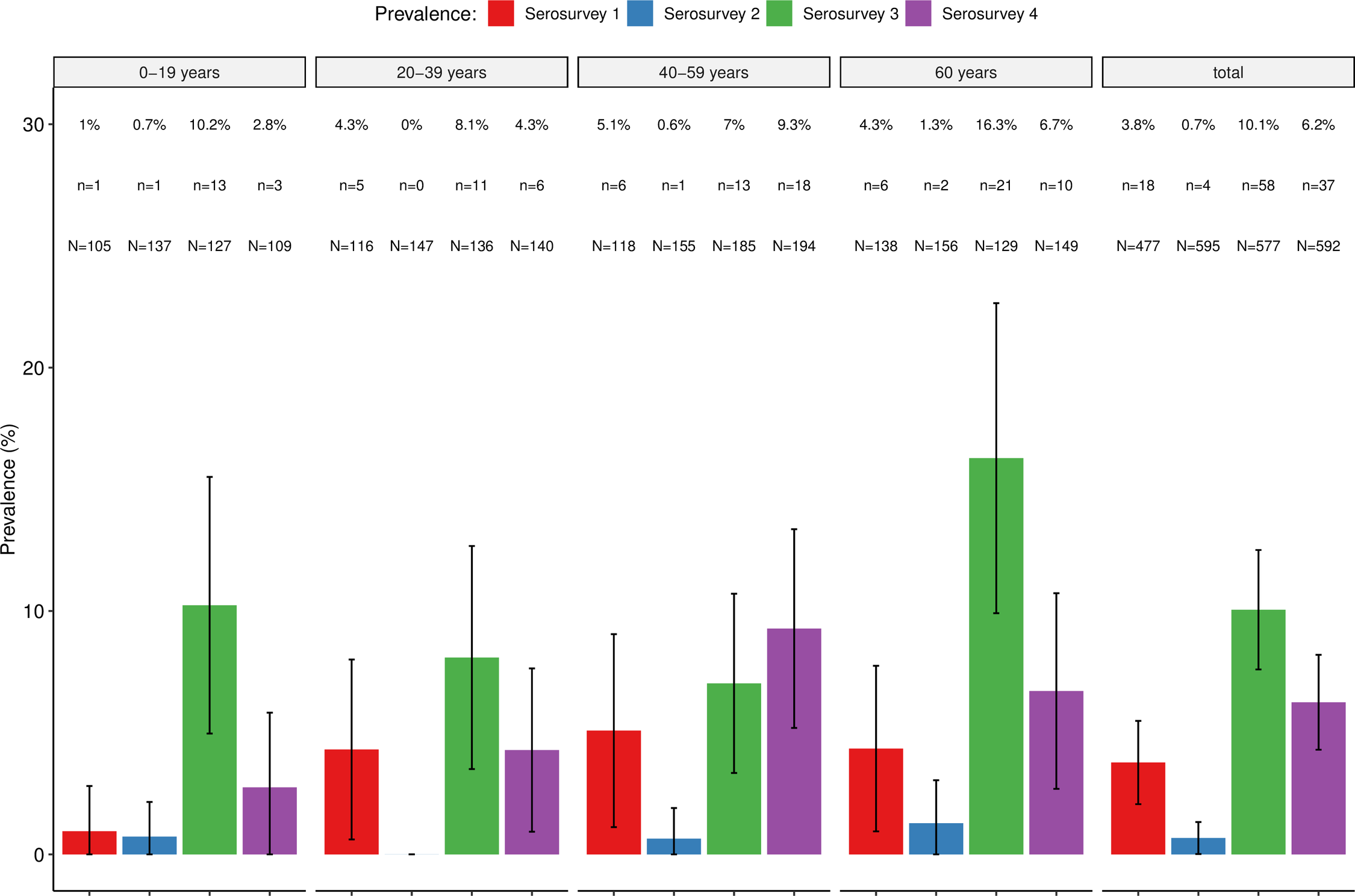

Supplement: Supplementary file 1 — Figure S1. Age‐specific prevalence of SARS‐CoV‐2 PCR‐positive infection in the sub‐sample of participants that provided a nasopharyngeal swab in the four community‐based seroepidemiological surveys, Manhiça district.%: prevalence of seropositives; n:absolute number of positives per age group; N:total sample size per age group. [file IRV-18-e13332-s001.tif]
