# Supplementary material for: High SARS‐CoV‐2 Exposure in Rural Southern Mozambique After Four Waves of COVID‐19: Community‐Based Seroepidemiological Surveys
Source: Influenza Other Respir Viruses. 2024 Jun 5;18(6):e13332. doi: 10.1111/irv.13332 (PMC11150860; doi:10.1111/irv.13332)
Supplement: Supplementary file 2 — Table S1. Age‐specific seroprevalence of SARS‐CoV‐2 antibodies in 4 community‐based seroepidemiological surveys in Manhiça district, southern Mozambique. Table S2. Seroprevalence of SARS‐CoV‐2 antibodies in 4 community‐based seroepidemiological surveys in Manhiça district, southern Mozambique in age groups not eligible for vaccination. Table S3. Comparison of seroprevalence of SARS‐CoV‐2 antibodies between age groups in 4 community‐based seroepidemiological surveys in the Manhiça district, southern Mozambique. Table S4. Evolution of the seroprevalence of SARS‐CoV‐2 antibodies by administrative post during 4 community‐based seroepidemiological surveys in Manhiça district. [file IRV-18-e13332-s002.docx]

**Supplementary Table 1. Age-specific seroprevalence of SARS-CoV-2 antibodies in 4 community-based seroepidemiological surveys in Manhiça district, southern Mozambique.**

|  | **Serosurvey - % (n/N)** | | | | | | | | χ^2^ or Fisher’s exact test ***p* values** | | |
| --- | --- | --- | --- | --- | --- | --- | --- | --- | --- | --- | --- |
|  | **Serosurvey 1** | | **Serosurvey 2** | | **Serosurvey 3** | | **Serosurvey 4** | | **Serosurvey 1 vs 2** | **Serosurvey 2 vs. 3** | **Serosurvey 3 vs 4** |
|  | % (95% CI) | n/N | % (95% CI) | n/N | % (95% CI) | n/N | % (95% CI) | n/N |  |  |  |
| **0 – 19 years** | 25.6 (18.9 – 32.2) | 43/168 | 58.2 (51.8 – 64.5) | 135/232 | 82.6 (77.0 – 88.2) | 147/178 | 87.6 (83.2 – 81.9) | 197/225 | <0.001 | <0.001 | 0.161 |
| **20 – 39 years** | 32.0 (25.2 – 38.8) | 58/181 | 64.4 (58.2 – 70.5) | 148/230 | 96.1 (93.3 – 98.9) | 173/180 | 94.3 (91.4 – 97.2) | 231/245 | <0.001 | <0.001 | 0.391 |
| **40 -59 – years** | 27.4 (20.4 – 34.4) | 43/157 | 66.8 (60.9 – 72.7) | 163/244 | 92.4 (88.9 – 96.7) | 206/223 | 94.2 (91.6 – 96.7) | 306/325 | <0.001 | <0.001 | 0.409 |
| **60 years or more** | 25.0 (18.3 – 31.7) | 40/160 | 64.8 (58.6 – 70.9) | 149/230 | 93.1 (89.4 – 96.7) | 174/187 | 87.9 (84.3 – 91.5) | 282/322 | <0.001 | <0.001 | 0.064 |
| **Total/overall** | 27.6 (24.2 – 31.0) | 184/666 | 63.6 (60.5 – 66.7) | 595/936 | 91.2 (89.1 – 93.2) | 700/768 | 91.1 (89.4 – 92.7) | 1017/1117 | <0.001 | <0.001 | 0.941 |

**Supplementary Table 2.** **Seroprevalence of SARS-CoV-2 antibodies in 4 community-based seroepidemiological surveys in Manhiça district, southern Mozambique in age groups not eligible for vaccination.**

| **Age group** | **Serosurvey - % (n/N)** | | | | | | | | χ^2^ or Fisher’s exact test ***p* values** | | |
| --- | --- | --- | --- | --- | --- | --- | --- | --- | --- | --- | --- |
|  | **Serosurvey 1** | | **Serosurvey 2** | | **Serosurvey 3** | | **Serosurvey 4** | | **Serosurvey 1 vs 2** | **Serosurvey 2 vs. 3** | **Serosurvey 3 vs 4** |
|  | % (95% CI) | n/N | % (95% CI) | n/N | % (95% CI) | n/N | % (95% CI) | n/N |  |  |  |
| 0 – 11 years | 16.9 (8.8 – 24.9) | 14/83 | 52.7 (44.1 – 61.2) | 69/131 | 77.1 (69.1 – 85.2) | 81/105 | 82.8 (76.3 – 89.3) | 106/128 | <0.001 | <0.001 | 0.279 |
| 12 – 17 years | 31.3 (19.9 – 42.6) | 20/64 | 60.9 (48.9 – 72.9) | 39/64 | 90 (82.4 – 97.6) | 54/60 | 93 (87.0 – 98.9) | 66/71 | 0.001 | <0.001 | 0.543 |

**Supplementary Table 3. Comparison of seroprevalence of SARS-CoV-2 antibodies between age groups in 4 community-based seroepidemiological surveys in the Manhiça district, southern Mozambique**

| **Serosurvey** | **Age group - % (n/N)** | | | | χ^2^ or Fisher’s exact test ***p* value** | | | | | |
| --- | --- | --- | --- | --- | --- | --- | --- | --- | --- | --- |
|  | **0 – 19 years** | **20 -39 years** | **40 – 59 years** | **60 or more years** | **0-19 vs. 20-39y** | **0-19 vs 40-59y** | **0-19 vs ≥60 y** | **20-39 vs 40-59y** | **20-39 vs ≥60 y** | **40-59 vs ≥60 y** |
| **Serosurvey 1** | 25.6 (43/168) | 32 (58/181) | 27.4 (43/157) | 25 (40/160) | 0.163 | 0.714 | 0.928 | 0.317 | 0.143 | 0.652 |
| **Serosurvey 2** | 58.2 (135/232) | 64.4 (148/230) | 66.8 (163/244) | 64.8 (149/230) | 0.174 | 0.052 | 0.145 | 0.574 | 0.922 | 0.643 |
| **Serosurvey 3** | 82.6 (147/178) | 96.1 (173/180) | 92.4 (206/223) | 93.1 (174/187) | **<0.001** | **0.003** | **0.002** | 0.115 | 0.196 | 0.795 |
| **Serosurvey 4** | 87.6 (197/225) | 94.3 (231/245) | 94.2 (306/325) | 87.9 (282/322) | **0.011** | **0.006** | 0.907 | 0.947 | **0.010** | **0.005** |

**Supplementary Table 4. Evolution of the seroprevalence of SARS-CoV-2 antibodies by administrative post during 4 community-based seroepidemiological surveys in Manhiça district.**

| **Administrative post** | **Serosurvey - % (n/N)** | | | | χ^2^ or Fisher’s exact test ***p* values** | | |
| --- | --- | --- | --- | --- | --- | --- | --- |
|  | **Serosurvey 1** | **Serosurvey 2** | **Serosurvey 3** | **Serosurvey 4** | **Serosurvey 1 vs 2** | **Serosurvey 2 vs. 3** | **Serosurvey 3 vs 4** |
| Manhiça-Sede | 23.4 (55/235) | 69.1 (248/359) | 90 (188/209) | 92.4 (338/366) | <0.001 | <0.001 | 0.322 |
| Maluana | 29.3 (36/123) | 54.1 (73/135) | 91.5 (118/129) | 89.9 (142/158) | <0.001 | <0.001 | 0.644 |
| 3 de Fevereiro | 28.2 (55/195) | 56.1 (120/214) | 91.1 (256/281) | 88.7 (346/390) | <0.001 | <0.001 | 0.316 |
| Xinavane | 38.6 (22/57) | 65.7 (69/105) | 92.3 (72/78) | 94.7 (108/114) | 0.002 | <0.001 | 0.495 |
| Ilha Josina Machel | 30.4 (7/23) | 78.4 (69/88) | 94 (47/50) | 90.6 (58/64) | <0.001 | 0.016 | 0.507 |
| Calanga | 27.3 (9/33) | 45.7 (16/35) | 90.5 (19/21) | 100 (25/25) | 0.137 | 0.001 | 0.203 |
